# Surface Structure Engineering of Nanosheet-Assembled NiFe_2_O_4_ Fluffy Flowers for Gas Sensing

**DOI:** 10.3390/nano11020297

**Published:** 2021-01-24

**Authors:** Xiaofeng Wang, Xu Li, Guozheng Zhang, Zihao Wang, Xue-Zhi Song, Zhenquan Tan

**Affiliations:** 1 Key Laboratory of Materials Modification by Laser Ion and Electron Beams, Dalian University of Technology, Ministry of Education, Dalian 116024, China; wangxf@dlut.edu.cn (X.W.); Lixu0114@163.com (X.L.); 15550320179@163.com (G.Z.); 2 State Key Laboratory of Fine Chemicals, Panjin Campus, School of Chemical Engineering, Dalian University of Technology, Panjin 124221, China; wangzh0621@mail.dlut.edu.cn

**Keywords:** gas sensor, NiFe_2_O_4_, nanosheet flowers, heating rate

## Abstract

In this work, we present a strategy to improve the gas-sensing performance of NiFe_2_O_4_ via a controllable annealing Ni/Fe precursor to fluffy NiFe_2_O_4_ nanosheet flowers. X-ray diffraction (XRD), a scanning electron microscope (SEM), nitrogen adsorption–desorption measurements and X-ray photoelectron spectroscopy (XPS) were used to characterize the crystal structure, morphology, specific surface area and surface structure. The gas-sensing performance was tested and the results demonstrate that the response was strongly influenced by the specific surface area and surface structure. The resultant NiFe_2_O_4_ nanosheet flowers with a heating rate of 8 °C min^−1^, which have a fluffier morphology and more oxygen vacancies in the surface, exhibited enhanced response and shortened response time toward ethanol. The easy approach facilitates the mass production of gas sensors based on bimetallic ferrites with high sensing performance via controlling the morphology and surface structure.

## 1. Introduction

Resistive gas sensors based on metal oxides, most of which are semiconductors (containing n-type and p-type semiconductors), have been widely used in a range of commercial gas detection systems [1]. Bimetallic semiconductor materials have stood out for gas sensing due to their low cost, easy manufacture, versatility, high stability, eco-friendliness and large range of detectable gases [2,3,4,5,6,7]. More interestingly, NiFe_2_O_4_ having an inverse spinel ferrite structure is characterized to be an n-type or p-type semiconductor which can be altered by controlling the stoichiometric and cation distribution [8,9,10,11]. Therefore, many efforts have been made to apply NiFe_2_O_4_ with various microstructures to the energy storage [12], catalysis [13] and effective detection of hazardous gases [14]. However, the gas-sensing properties of the NiFe_2_O_4_-based sensor are expected to be further enhanced. Regulating the surface active sites and microstructures of NiFe_2_O_4_ is considered to be an effective method as the gas-sensing process involves the surface reaction.

For one thing, it is widely acknowledged that a large specific surface area can increase the number of active atoms which are able to participate in the gas-sensing process [15,16]. Designing a microstructure of NiFe_2_O_4_ with less agglomeration, large specific surface area and good surface permeability is a promising approach to enhance the gas-sensing performance [17,18]. Using a carbon sphere as a template, Zhou et al. prepared NiFe_2_O_4_ nanospheres with a core-in-shell structure as a gas-sensing material [19]. The homologous sensor exhibited rapid response and recovery to 100 ppm acetone with a response value of 10.6. Zhang et al. successfully synthesized NiFe_2_O_4_ nano-octahedrons via pyrolysis of Ni/Fe-bimetallic metal–organic frameworks (MOFs), and the as-fabricated sensor exhibited immense potential in monitoring toluene gas [20]. Our group has also conducted several research works on nanostructured bimetallic ferrites including hollow NiFe_2_O_4_ materials, which were synthesized through annealing treatment of MOFs and exhibited good gas-sensing performances including high response, good selectivity and low working temperature [21,22]. However, the synthetic strategies of these porous or hollow structured materials are usually carried out by template-directed preparation, a tedious method with material waste and low yield [23,24]. Thus, it is of great significance to develop a facile and scalable route for the synthesis of materials with a large specific surface area.

Additionally, defects in semiconductor materials can adjust the conductivity and surface activity of materials, which are critical factors in influencing the gas-sensing response depending on the variation of resistance [25]. According to some literature studies, the ubiquitous oxygen vacancy in the materials can improve gas-sensing characteristics [26,27].

As a proof of concept, we herein propose an easy approach to generate NiFe_2_O_4_ fluffy nanosheet-assembled flowers (NSFs) with an enlarged specific surface area and increased concentration of oxygen vacancies arising from the heating rate control. The synergistic effect of larger specific surface area, fluffy feature and more oxygen vacancies led to better gas-sensing performances toward ethanol gas including higher sensitivity and faster response. This facile approach offers guidance on designing superior gas sensors based on semiconducting metal oxides via morphology and defect engineering. The gas-sensing results are helpful in understanding the gas-sensing mechanism.

## 2. Materials and Methods

### 2.1. Preparation of NiFe_2_O_4_ NSFs

NiFe_2_O_4_ NSFs were synthesized by a facile one-step hydrothermal process and subsequent annealing treatment. Briefly, a homogenous solution of Ni(NO_3_)_2_·6H_2_O (1 mmol), Fe(NO_3_)_3_·9H_2_O (0.67 mmol), NH_4_F (2.67 mmol), CO(NH_2_)_2_ (4 mmol) and 30 mL deionized water was heated in a Teflon-lined autoclave for 6 h at 100 °C. After being washed with deionized water and ethanol for three times and dried, the yellow sediment was annealed at 500 °C for 3 h to obtain NiFe_2_O_4_ NSFs with a heating rate of 2, 5 and 8 °C min^−1^, respectively, denoted as NFO-2, NFO-5 and NFO-8 NSFs.

### 2.2. Materials Characterization

The crystal structure, morphology and surface property of NiFe_2_O_4_ were characterized with XRD, SEM, transmission electron microscopy (TEM), nitrogen adsorption–desorption measurement and XPS. The information of the testing equipment and testing condition can be found in the Supplementary Information.

### 2.3. Fabrication and Measurement of Gas Sensor

The gas sensor was fabricated and measured following our previous works [3,7]. The as-synthesized NiFe_2_O_4_ NSFs were coated on the outside surface of an alumina ceramic tube with a pair of Au electrodes at each end and a heating coil inside. The gas-sensing measurement was performed on a CGS-8 System (Beijing Elite Technology Co., Ltd., Beijing, China). A schematic diagram of the sensing setup and sensing device is shown in Figure S1. The response of a sensor is generally defined as *R*_g_/*R*_a_, where *R*_g_ and *R*_a_ represent the sensor resistance in the target gas and air, respectively.

## 3. Results

### 3.1. Structures Characterization

**Figure 1:**
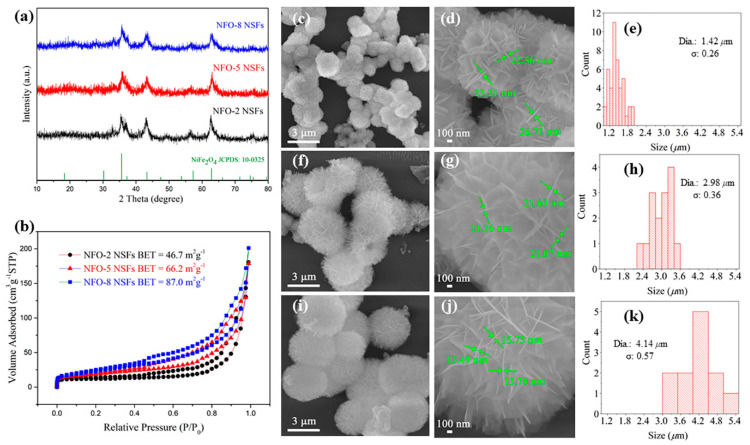
The XRD patterns (**a**) and nitrogen adsorption–desorption isotherms and Brunner–Emmet–Teller (BET) surface area values (**b**) of NFO-2, NFO-5 and NFO-8 nanosheet-assembled flowers (NSFs); SEM images and flower size distribution of NFO-2 NSFs (**c**–**e**), NFO-5 NSFs (**f**–**h**) and NFO-8 NSFs (**i**–**k**).

Based on the XRD patterns of the synthesized NiFe_2_O_4_ NSFs with a heating rate of 2, 5 and 8 °C min^−1^, respectively, denoted as NFO-2, NFO-5 and NFO-8 NSFs (shown in Figure 1a), it was found that all three as-prepared samples matched well with the monoclinic NiFe_2_O_4_ phase, indexed to JCPDS: 10-0325. Nitrogen adsorption–desorption isotherms and Brunner–Emmet–Teller (BET) surface area values of the synthesized NiFe_2_O_4_ NSFs are presented in Figure 1b. A higher heating rate of 8 °C min^−1^ endows the NiFe_2_O_4_ with increased BET surface area (87.0 m^2^ g^−1^) compared with the samples acquired under 2 °C min^−1^ and 5 °C min^−1^ (BET surface areas: 46.7 m^2^ g^−1^ and 66.2 m^2^ g^−1^). The larger specific surface area of NFO-8 NSFs can provide more active sites to adsorb oxygen species and react with target gas molecules, which can facilitate the gas-sensing performances.

To explore the morphologies of the NiFe_2_O_4_ NSFs prepared at different heating rates, the SEM images and particle size distribution are gathered in Figure 1c–k. The NiFe_2_O_4_ nanosheets self-assembled together, forming nanosheet flowers. The sizes of the flowers formed under the heating rate of 2, 5 and 8 °C min^−1^ were ca. 1.42, 2.98 and 4.14 μm, respectively. As the three NiFe_2_O_4_ samples came from the same precursor, the NFO-8 NSFs clearly possessed the largest size of the flower, then a large specific surface area was formed, which was in accordance with the results of BET. The nanosheets of NFO-8 NSFs were ultrathin and the flowers were fluffier. The corresponding TEM image in Figure S2a also reveals the ultrathin NiFe_2_O_4_ nanosheets in the NFO-8 NSFs. Moreover, the representative HRTEM image in Figure S2b shows a set of lattice fringes with the interplanar distance of 0.25 nm which resulted from the (311) facets of spinel NiFe_2_O_4_.

**Figure 2:**
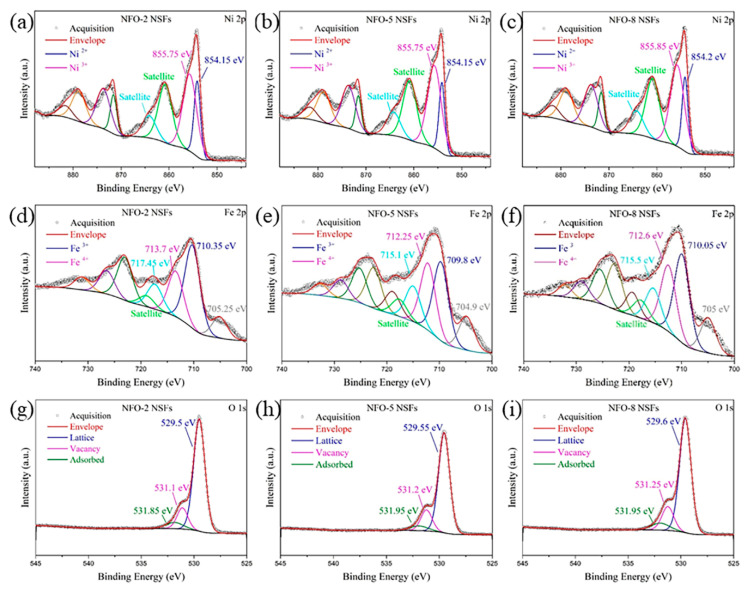
XPS Ni 2p spectra of (**a**) NFO-2, (**b**) NFO-5 and (**c**) NFO-8 NSFs; XPS Fe 2p spectra of (**d**) NFO-2, (**e**) NFO-5 and (**f**) NFO-8 NSFs; XPS O 1s spectra of (**g**) NFO-2, (**h**) NFO-5 and (**i**) NFO-8 NSFs.

To characterize the surface defects of NiFe_2_O_4_ NSFs, the XPS measurements were conducted which could provide some information of the elemental valence state and the surface characteristics. The well-resolved Ni 2p spectra in Figure 2a–c exhibit two main peaks at ca. 854.15 and 855.75 eV for Ni^2+^ and Ni^3+^. The two pairs of coupled peaks in Figure 2d–f with binding energies at ca. 710.05 and 712.60 eV correspond to the binding energy of the Fe^3+^ and Fe^4+^ [28]. The ratios of Ni^3+^/Ni^2+^ in NFO-2, NFO-5 and NFO-8 NSFs are 2.22, 2.80 and 2.56 and the ratios of Fe^4+^/Fe^3+^ are 0.54, 0.85 and 0.75, respectively. The amounts of Ni^3+^ and Fe^4+^ in NFO-5 and NFO-8 NSFs were much more than those in NFO-2 NSFs, which means more defects in the materials of the NFO-5 and NFO-8 NSFs. The relative contents of oxygen defects can be analyzed and calculated from the O 1s peak in Figure 2g–i. From low to high banding energy, the resolved peaks at ca. 529.5, 531.2 and 531.9 eV are usually associated with the metal–oxygen bonding (O_L_), oxygen vacancy-related defects (O_V_) and absorbed oxygen (O_abs_), respectively [29,30,31]. The relative contents of O_L_, O_V_ and O_abs_ calculated from the O 1s peak fitting of NFO-2, NFO-5 and NFO-8 NSFs are summarized in Table S1. Obviously, the NFO-5 and NFO-8 NSFs possess higher contents of O_V_ and O_abs_ than NFO-2 NSFs, which play critical roles in the resistance and response of NiFe_2_O_4_ sensors.

### 3.2. Ethanol Sensing Performance

**Figure 3:**
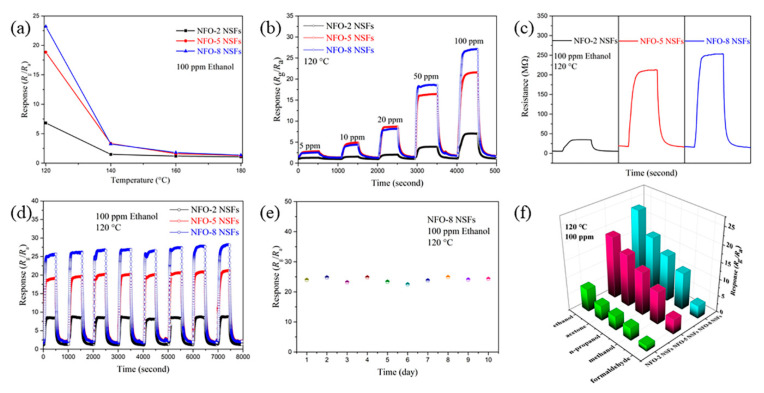
(**a**) Gas-sensing response versus operating temperature of the sensors upon exposure to 100 ppm ethanol; (**b**) the response versus time of the sensors upon exposure to ethanol ranging from 5 to 100 ppm; (**c**) the resistance transient of the sensors; (**d**) the reproducibility testing of the sensors; (**e**) the long-term stability of the NFO-8 NSFs-based sensor measured for 10 days. (**f**) Comparison of the responses of the sensors for various gases.

The sensing performance of the NiFe_2_O_4_ NSFs was evaluated by separately detecting different volatile organic compounds (VOCs) including ethanol (C_2_H_5_OH), acetone (C_3_H_6_O), n-propanol (C_3_H_7_OH), methanol (CH_3_OH) and formaldehyde (HCHO). Firstly, we determined the optimal operating temperature of all the three sensors by monitoring the real-time electrical resistances in air and under 100 ppm ethanol at the temperature range of 120–180 °C (shown in Figure 3a). For each sample, the highest response toward 100 ppm ethanol appeared at 120 °C, and the response decreases as the operating temperature elevates. It is worth mentioning that the test at the temperature lower than 120 °C is meaningless as the electrical resistances in air of all the three sensors fail to achieve steady values. Therefore, 120 °C was selected as the operating temperature in subsequent tests. The changes in gas-sensing responses of NFO-2, NFO-5 and NFO-8 NSFs over gas concentration were investigated during 5 to 100 ppm ethanol gas (Figure 3b). At the concentrations of 50 and 100 ppm, the NFO-8 NSFs-based sensor showed the highest response values compared with the NFO-2 and NFO-5 NSFs-based sensors. Meanwhile, at the concentration range of 5–20 ppm, the NFO-8 NSFs- and NFO-5 NSFs-based sensors showed approximately equal responses, which are much higher than that of the NFO-2 NSFs-based sensor. The improvement in the sensing response of the NFO-8 NSFs-based sensor toward ethanol gas is mainly attributed to the combined effect of the fluffy structure and surface composition. The fluffy structure with a higher specific surface area can expose more active sites for sensing reaction and facilitate the diffusion of ethanol molecules. Moreover, the oxygen vacancies in the material surface act as electron donors providing unpaired electrons and an active site which can improve the gas-sensing performances [32]. Although the calculated contents of oxygen vacancies of NFO-5 NSFs and NFO-8 NSFs are similar, the specific surface area of NFO-5 NSFs is much lower than that of NFO-8 NSFs. Thus, the larger specific surface area and fluffy features of NFO-8 NSFs with oxygen vacancies undoubtedly provide more active sites for the adsorption of target gas molecules and oxygen species.

To more deeply understand the properties related to the gas-sensing performance of the three sensors, the dynamic sensing transients of resistances were tested. As shown in Figure 3c, the resistances clearly increase in ethanol gas and decrease in air, indicating the p-type semiconductor conductivity, which is due to the hole hopping between Ni^3+^ and Ni^2+^ [33]. In addition, it can be noted that the resistance of samples in air with a relatively higher heating rate (5 and 8 °C min^−1^) was much larger than that of 2 °C min^−1^. This can be ascribed to their fluffier structure and the decreasing carrier concentration. As the structure becomes fluffier, fewer electric channels can be produced in the materials of NFO-5 and NFO-8 NSFs. In addition, free electrons produced by oxygen vacancies compensate part of the hole carrier and increase the resistance in the air of p-type NFO-5 and NFO-8 NSFs [34,35].

The response time (*τ*_res_) of the sensors was calculated from Figure S3 to be 141, 120 and 104 s for NFO-2, NFO-5 and NFO-8 NSFs, respectively. The recovery time (*τ*_rec_) was 113, 105 and 77 s for NFO-2, NFO-5 and NFO-8 NSFs, respectively. The response and recovery were not very quick which may be due to the relatively low working temperature of 120 °C [32]. Even so, the response/recovery process of the sensors based on NFO-8 NSFs is faster compared to the other two samples, which might benefit from the easy diffusion of gas molecules due to the fluffy structure with a high specific surface area. The responses of the NFO-8 NSFs-based sensor to 100 ppm ethanol, acetone, n-propanol, methanol and formaldehyde were 23.2, 17.1, 13.9, 11.1 and 3.9 at 120 °C, respectively (summarized in Figure 3f). The results indicate a selectivity toward ethanol gas. The different lowest unoccupied molecule orbit (LUMO) energy for various target gas molecules leads to different responses. Moreover, the selectivity toward ethanol gas could be ascribed to the strong interaction between the ethanol molecules and the surface of NFO-8 NSFs at 120 °C [36]. Our material can be used as part of a sensor array to provide a response pattern.

The stability of gas sensors is a crucial factor for practical application. The as-fabricated sensors exhibited satisfactory cyclic stability and repeatability as the response values were roughly constant for eight successive cycles alternatively exposed to air and 100 ppm ethanol gas (Figure 3d). Furthermore, the sensor based on NFO-8 NSFs also showed a satisfactory long-term stability with a slight variation in response towards 100 ppm ethanol gas during the 10 days (Figure 3e).

### 3.3. Gas-Sensing Mechanism

For resistance-type gas-sensing material, the wildly accepted reason for the resistance change during the testing process is the electron gain and loss model. At an air atmosphere, oxygen molecules are pre-adsorbed on the surface of gas-sensing materials, forming active oxygen species by capturing free electrons [37,38,39,40,41,42]. Simultaneously, the concentration of the hole carrier in the p-type semiconductor increases, resulting in a resistance decrease. When ethanol gas is introduced, the ethanol molecules will react with the pre-absorbed oxygen species or directly adsorb on the surface [25]. The reaction with the pre-absorbed oxygen species will release the captured electrons back to the gas-sensing materials, increasing the resistance. Further, the direct adsorption will be accompanied by a charge transfer from the ethanol molecules to the surface, increasing the resistance of p-type semiconductor materials [43,44,45].

In this work, the improvement in the ethanol-sensing performance of NFO-8 NSFs is mainly attributed to the synergy effect of the fluffy structure and surface composition. On one hand, the high specific surface area and fluffy structure of NFO-8 NSFs expose more active sites for the sensing reaction and facilitate the diffusion of ethanol molecules, accelerating the process of gas adsorption–desorption. On the other hand, the oxygen vacancies in the material surface can lower the adsorption energy, modify the electronic state of metal cations and provide active sites for the gas-sensing process. [32,46,47,48] Thus, the larger surface area and fluffy structure of NFO-8 NSFs with oxygen vacancies undoubtedly enhance the reaction between the target gas and the surface.

## 4. Conclusions

In summary, fluffy NiFe_2_O_4_ nanosheet flowers labeled as NFO-2, NFO-5 and NFO-8 NSFs have been successfully prepared by a facile one-step hydrothermal approach after annealing at 500 °C for 3 h with a heating rate of 2, 5 and 8 °C min^−1^, respectively. Compared with the NFO-2 NSFs- and NFO-5 NSFs-based sensors, the p-type semiconducting NFO-8 NSFs-based sensor can present enhanced ethanol-sensing behavior at a low working temperature (120 °C), which exhibits a response of 23.2 to 100 ppm ethanol, benefiting from the fluffy structure, high specific surface area and surface oxygen vacancies. This facile and effective approach opens up a perspective for mass production, miniaturization and commercialization of the relevant sensors.

## Supplementary Materials

The following are available online at https://www.mdpi.com/2079-4991/11/2/297/s1, Figure S1: Schematic diagram of the sensing setup and sensing device, Figure S2: TEM characterization of NFO-8 NSFs; (b) HRTEM image of NFO-8 NSFs, Figure S3: Response time and recovery time of the sensor towards 100 ppm ethanol at 120 °C, Table S1: The contents of O in different forms summarized from O 1s XPS results of the NiFe_2_O_4_ NSFs.
