# Supplementary material for: Surface Structure Engineering of Nanosheet-Assembled NiFe2O4 Fluffy Flowers for Gas Sensing
Source: Nanomaterials (Basel). 2021 Jan 24;11(2):297. doi: 10.3390/nano11020297 (PMC7911288; doi:10.3390/nano11020297)
Supplement: Supplementary file 1 [file nanomaterials-11-00297-s001.pdf]

nanomaterials

Supplementary Materials

Surface Structure Engineering of Nanosheet-Assembled $NiFe_2O_4$ Fluffy Flowers for Gas Sensing

Xiaofeng Wang [1], Xu Li [1], Guozheng Zhang [1], Zihao Wang [2], Xue-Zhi Song [2,*] and Zhenquan Tan [2,*]

1 Key Laboratory of Materials Modification by Laser Ion and Electron Beams, Dalian University of Technology, Ministry of Education, Dalian 116024, China
2 State Key Laboratory of Fine Chemicals, School of Chemical Engineering, Dalian University of Technology, Panjin Campus, Panjin 124221, China
* Correspondence: songxz@dlut.edu.cn (X.-Z.S.), tanzq@dlut.edu.cn (Z.T.)

1. Materials characterization

The crystal structure of $NiFe_2O_4$ was characterized using a SHIMADZU XRD-7000S X-ray diffraction with Cu $K\alpha_1$ radiation (λ=1.5406 Å). A Quantachrome Autosorb iQ-1C TCD surface area analyzer was applied to carry out the nitrogen adsorption-desorption measurements at 77 K after degassing under vacuum at 300 °C for 3 h. A FEI Nova NanoSEM 450 field-emission scanning electron microscope was used to investigate the morphologies of the $NiFe_2O_4$ samples. Transmission electron microscopy (TEM) and high-resolution TEM (HRTEM) images were observed by transmission electron microscope (FEI Tecnai-G^{2}F30) at an acceleration voltage of 200 kV to characterize the morphology and microstructures. X-ray photoelectron spectroscopy (XPS) spectra were performed under Al $K\alpha$ X-ray radiation on a ThermoFisher ESCALAB™ 250Xi equipment, which were calibrated by a C 1s peak (284.6 eV).

2. Fabrication and measurement of gas sensor

A homogeneous slurry was formed by dispersing the as-synthesized $NiFe_2O_4$ NSFs in proper amount of deionized water, which was subsequently coated on the outside surface of an alumina ceramic tube with a pair of Au electrodes at each end. After drying, a Ni-Cr alloy coil was inserted into the alumina ceramic tube, that can control the operating temperature by changing the current flowing through the coil. The gas sensor was complete after welding the combination of heater and alumina tube coated with $NiFe_2O_4$ NSFs on a pedestal. The gas-sensing measurement was performed on a commercial CGS-8 Intelligent Gas Sensing Analysis System (Beijing Elite Technology Co., Ltd, China). Schematic diagram of the sensing set-up and sensing device were shown in Fig. S1. The response of a sensor based on a typical p-type semiconductor material is generally defined as R_g/R_a, where R_g and R_a represent the resistance of the sensor in target gases and in air, respectively. Furthermore, the response time (τ_{res}) and recovery time (τ_{rec}) were evaluated as the time for 90% of the resistance change in adsorption and desorption processes. Three sensors were tested for each materials, and the one whose sensing performance was close to the average performance was used as the final testing device.

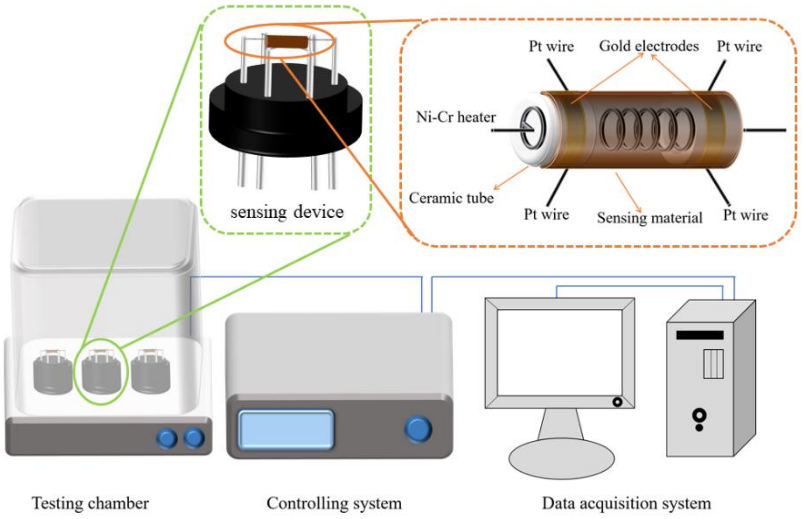

Figure S1. Schematic diagram of the sensing set-up and sensing device.

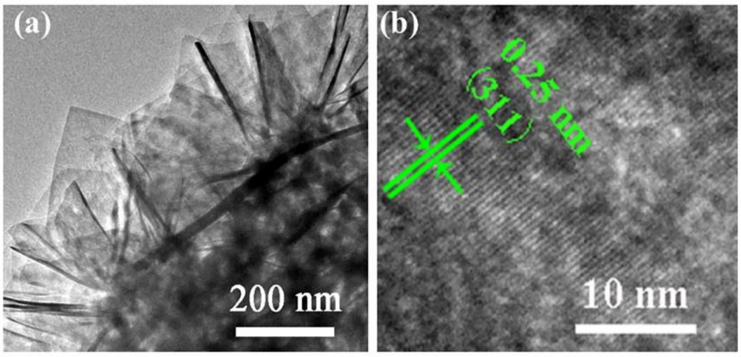

Figure S2. TEM characterization of NFO-8 NSFs; (b) HRTEM image of NFO-8 NSFs.

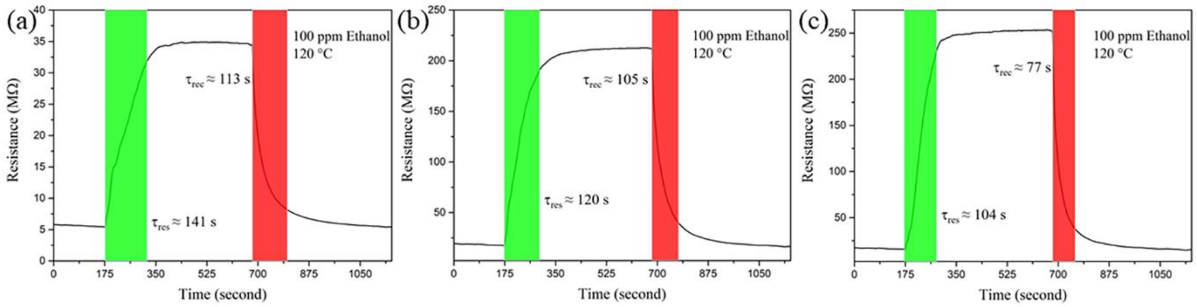

Figure S3. Response time and recovery times of the sensors based on (a) NFO-2, (b) NFO-5 and (c) NFO-8 NSFs toward 100 ppm ethanol at 120 °C.

Table 1. The contents of O in different forms summarized from O 1s XPS results of the $NiFe_2O_4$ NSFs.

**Materials**	**O_L**	**O_V**	**O_{abs}**
NFO-2	529.50 (79%)	531.10 (14%)	531.83 (7%)
NFO-5	529.55 (77%)	531.21 (17%)	531.90 (6%)
NFO-8	529.58 (76%)	531.23 (17%)	531.93 (7%)
